# The Different Immunoregulatory Functions of Mesenchymal Stem Cells in Patients with Low-Risk or High-Risk Myelodysplastic Syndromes

**DOI:** 10.1371/journal.pone.0045675

**Published:** 2012-09-21

**Authors:** Zhigang Zhao, Zhenling Wang, Qiubai Li, Weiming Li, Yong You, Ping Zou

**Affiliations:** 1 Department of Hematology, The Oncology Hospital of Tianjin Medical University, Tianjin, P.R. China; 2 Department of Hematology, Institute of Hematology, Tongji Medical College of Huazhong University of Science and Technology, Wuhan, P.R. China; University of Medicine and Dentistry of New Jersey, United States of America

## Abstract

Myelodysplastic syndrome (MDS) are a group of progressive, clonal, neoplastic bone marrow disorders characterized by hematopoietic stem cell dysregulation and abnormalities in the immune system. Mesenchymal stem cells (MSC) have gained further interests after the demonstration of an immunoregulatory role. Nevertheless, the immunoregulatory function of MDS bone marrow derived MSC (MDS-MSC) remains poorly defined. In addition, it is not clear whether there are differences in the regulatory functions between low-risk and high-risk MDS-MSC. In this study, we obtain and expand MSC from bone marrow of patients with MDS. Our results show that there are significant differences in the immunoregulatory functions between low-risk and high-risk MDS-MSC. Compare to low-risk MDS-MSC, high-risk MDS-MSC is associated with the presence of increased TGF-β1, higher apoptosis, higher immunosuppressive rate and a poor ability of hematopoietic support. In addition, our results find that there are great differences in the CD4+CD25+Foxp3+Tregs inducible rate between high-risk MDS-MSC and low-risk MDS-MSC. Compared to high-risk MDS-MSC, the inducible rate of CD4+CD25+Foxp3+Tregs of low-risk MDS-MSC is lower. At last, we find that MDS-MSC derived TGF-β1 is largely responsible for the increase in CD4+CD25+Foxp3+Tregs based on knockdown studies. These results elucidate the different immunoregulatory role of MSC in low-risk and high-risk MDS, which may be important for understand the pathogenesis of MDS and the development of novel immunomodulatory strategies for the treatment of MDS.

## Introduction

Myelodysplastic syndrome (MDS) comprise a heterogeneous group of clonal hematopoietic stem cell malignancies characterized by ineffective bone marrow (BM) hematopoiesis, peripheral blood cytopenias and substantial risk for progression to acute myeloid leukemia. Several clinical and immunological studies suggest a significant deregulation of the immune system in the complex pathogenesis of MDS. This deregulation may even promote the progression of early MDS to advanced MDS [1]–[2]. In addition, BM failure in partial early or low-risk MDS patients has been considered to be related to the strong immunologic function of T lymphocytes. Consistent with it, previous studies had confirmed that immunosuppressive therapy with ATG or CSA could lead to lasting hematologic responses and abrogation of T-cell clones, which was particularly noticeable in low-risk MDS [3]–[4].

Using the International Prognostic Scoring System (IPSS), MDS can be broadly separated into low-risk (IPSS score≤1.0) and high-risk (IPSS score>1.0), each with distinct pathology [5]. There are several differences between low-risk and high-risk MDS. First, compared to high-risk MDS, low-risk MDS have a good prognosis and longer survival. Second, low-risk MDS is characterized by increased apoptosis in the bone marrow with autoimmune characteristics whereas the high-risk stages involve immune evasion and cytogenetic abnormalities, giving cells growth potential to progress into leukemia. At last, there are big differences in the immune abnormalities between low-risk and high-risk MDS. Compare to low-risk MDS, high-risk MDS is associated with the presence of dysfunctional NK cells, increased Tregs, increased cytotoxic CD8+ T cells, lower apoptosis and a poor response to immunosuppressive therapy. All these differences show that low-risk MDS and high-risk MDS possess different biological characteristics [6].

As we all know, mesenchymal stem cells (MSC) have immunomodulatory properties, exerted by cell-to-cell contact and in a paracrine fashion [7]–[8]. Previous studies have shown that MSC are inherently low of immunogenicity and are capable of inhibiting T cells proliferation *in vitro* and mediate a systemic immunosuppressive property *in vivo* [9]–[10]. In addition, MSC represent an important cellular component in the bone marrow microenvironment. This raised the question of whether MSC play a certain role in the immune abnormalities and the pathogenesis of MDS.

Our previous studies demonstrated that although MDS bone marrow derived MSC (MDS-MSC) were similar to normal adult bone marrow derived MSC in morphology, growth property, surface epitopes, and differentiation ability *in vitro*, the immunoregulatory functions of MDS-MSC were impaired, suggesting the involvement of MSC in the pathogenesis of MDS [11]. In addition, the different immune abnormalities between low-risk and high-risk MDS prompted us to investigate whether there were differences in the immunoregulatory functions between low-risk and high-risk MDS-MSC. Moreover, it is unclear what is the definitive role of MSC in the pathogenesis of MDS with different phase.

The current study is designed to investigate the immunoregulatory functions of low-risk and high-risk MDS-MSC. Our data show that there is a big difference in the immune abnormality between low-risk and high-risk MDS-MSC. Compare to low-risk MDS-MSC, high-risk MDS-MSC is associated with the presence of increased Tregs, increased TGF-β1, higher apoptosis, higher immunosuppressive rate and a poor ability of hematopoietic support. In addition, our results demonstrate that MDS-MSC derived TGF-β1 is largely responsible for the increase in CD4+CD25+Foxp3+Tregs based on knockdown studies.

## Materials and Methods

### Patient Characteristics

Fourteen patients with low-risk MDS (aged from 38 to 56; IPSS score≤1.0) and fifteen patients with high-risk MDS (aged from 32 to 57; IPSS score>1.0) were investigated in this study; Ten healthy donors (aged from 30 to 55) were also recruited. All patients were in their initial diagnose and untreated at the time of study. Diagnosis was established by BM aspirate smears, BM biopsy, cytogenetic analyses and peripheral blood count criteria, according to the WHO group.

### Isolation and Culture of MSC

After receiving informed consent from the patients according to the academic guidelines on the use of human subjects in research, human bone marrow was obtained from patients and healthy donors under the protocol approved by the institutional review board. Mononuclear cells (MNC) were separated by a Ficoll-Paque gradient centrifugation (specific gravity 1.077 g/ml; Sigma Diagnostics, St Louis, MO, USA), and cultured in expansion medium at 37°C with 5% CO_2_ in fully humidified atmosphere. Expansion medium contained 60% DMEM/F-12 (Gibco Life Technologies, Paisley, UK), 40% MCDB-201 (Sigma), 2% fetal calf serum (FCS; Gibco), 1× insulin transferrin selenium, 1 × linoleic acid bovine serum albumin, 10^−9^M dexamethasone (sigma), 5 ng/ml basic fibroblast growth factor (Gibco), 10 ng/mL platelet-derived growth factor BB (PDGF-BB; Sigma), 10 ng/mL bone morphogenetic protein-4 (BMP-4; Sigma), 10 ng/mL insulin like growth factor (IGF; Sigma), 100 U/mL penicillin, 1000 U/mL streptomycin (Gibco). After culture for 24–48 h, the culture medium was replaced and non adherent cells were removed. Once cells were more than 80% confluent, they were detached with 0.25% trypsin-EDTA (Sigma), then CD14 positive cells were depleted using CD14 micromagnetic beads (Miltenyi Biotec, Auburn, USA).and CD14 negative cells were replated. To ensure single cell originality of each cell colony, sorted cells were plated at concentrations of 1 cells/well in 96 well plate coated by fibronectin (Sigma) in each setting and cultured in expansion medium. Wells with single adherent cell were identified during the first 24 hours. The appearance of cell colonies was checked daily. Single colony was harvested by trypsinization and expanded [12].

### Preparation of T Cell Subsets

Human peripheral blood mononuclear cells (hPBMC) from healthy donors were isolated by centrifugation over Ficoll-Hypaque gradients (Nycomed Amersham, Uppsala, Sweden). CD4+ T lymphocytes were isolated from hPBMC by using CD4 micromagnetic beads (Miltenyi Biotec, Auburn, USA) according to the manufacturer’s instruction. CD4+ cell purity was 97% ±2%. Subsequently, CD4+CD25-T cells and CD4+CD25+T cells were obtained by using the CD25 beads (Miltenyi Biotec).

### Coculture Experiment

MSC (either normal-MSC or MDS-MSC or low-risk MDS-MSC or high-risk MDS-MSC) and T cells (either CD4+CD25-T cells or CD4+CD25+T cells) were cocultured at 1∶1 ratio at 10^6^ cells/mL in RPMI 1640 with 5% FCS.

### FACS Analysis

For immunophenotype analysis, cultured cells were washed with PBS containing 0.5% bovine serum albumin (BSA, Sigma), and incubated with primary antibodies (10–20 ng/ml) for 30 minutes at 4°C. Primary antibodies included mAb against CD4, CD25, CD40, CD80, CD86 and Foxp3 (BD Biosciences Pharmingen, San Diego, CA, USA). We used same-species, same-isotype irrelevant antibody as negative control. Cell analysis was performed with FACS Calibur system using Cellquest software.

### Cytokine Analysis

Cytokines produced in culture supernatants at day 3 from MSC were detected by using ELISA kits (R&D Systems) for interleukin (IL)-3, IL-6, IL-11, transforming growth factor β1 (TGF-β1) and hepatocyte growth factor (HGF)1.

### Mixed Leukocyte Reaction

Allogeneic CD2+ T cells were purified from hPBMC by using the MACS CD2 isolation kit. CD2+T cells resuspended at 1×10^5^ to 1×10^6^ cells/well were added to wells containing or lacking irradiated (15 Gy) allogeneic suppressive cells. The culture was continued and 3H-thymidine was added 18 hours before the end of the 120-hour culture. The T-cell proliferation was represented as the incorporated radioactivity in cpm and shown as mean±SD of triplicate values.

### Effect of MSC on T Cell Apoptosis

MSC and MNC were prepared as described before. T cells were cultured alone or cocultured with MSC with PHA (5 µg/ml) stimulation for 3 days, then harvested and quantified, stained with Annexin-V kit (BD,USA), and analyzed by flow cytometry.

### TGF-β1 Knockdown in MSC

TGF-β1 siRNA duplexes were used to knock down this respective gene in MSC. Briefly, MSC (10^6^) were seeded in 75-cm^2^ flasks, after 24 h, 100 nM siRNA was delivered via DharmaFECT Transfection Reagent (Dharmacon, USA). TGF-β1 siRNA duplex sequence was selected as follows: 5′-gca aca auu ccu ggc gau a-3′ and 5′-uau cgc cag gaa uug uug c-3′. All siRNA duplexes were obtained from Takara Biotech (Dalian, China). Knockdown of TGF-β1 was confirmed by western blot.

### Western Blotting

For Western blotting, equivalent amount of protein lysates, obtained from induced cells, were loaded per lane. After SDS-PAGE, proteins were electrophoretically transferred onto nitrocellulose membrane (Amersham Pharmacia Biotech; Uppsala, Sweden). After blocking, blots were incubated with mouse polyclonal antibodies against TGF-β1 (Santa Cruz Biotechnology) at 1∶200. Expression of the β-actin was used as an internal control. Immunodetection using the enhanced chemiluminescence method (ECL kit; Amersham, Piscataway, N.J.) was performed according to the manufacturer’s instructions.

### Statistical Analysis

The results were statistically analyzed by using the SPSS11.0 statistical package (SPSS Inc, Chicago, IL). The Student t test for paired data (2-tail) and ANOVA was used to test the probability of significant differences between samples.

## Results

### Biological Characteristics of MDS-MSC

In this study, MSC were obtained from 14 low-risk MDS and 13 high-risk MDS patients’ bone marrow. We failed to isolate adherent cells from two high-risk MDS patients. These patients, aged 41 and 46 respectively, were all male at diagnosis of MDS-RAEB-1. Eighty-four clones,came from twenty-seven different patients, were obtained and analyzed for their immunoregulatory characteristics. MSC derived from MDS and normal adult displayed homogeneous fibroblast-like morphology. Two weeks later, almost all obtained MSC formed colony-forming unit fibroblast colonies (CFU-F). The frequency of CFU-F were 6.24±1.21 (per 10^6^ bone marrow mononuclear cells; n = 12) for high-risk MDS-MSC, and 7.39±0.85 (per 10^6^ bone marrow mononuclear cells; n = 14) for low-risk MDS-MSC, and 7.16±0.62 (per 10^6^ bone marrow mononuclear cells; n = 10) for normal-MSC, respectively. In addition, previous studies demonstrated that human MSC did not express costimulatory molecules B7-1, B7-2, CD40, and CD40 ligand [13]. So, we examine the immunophenotype of MDS-MSC. Our results demonstrated that these cells were negative for the expression of CD80, CD86, CD40 and HLA-DR. The immunophenotype of MSC remained unchanged during culture-expansion for at least 20 passages.

### Cytokine Secreted by MDS-MSC

**Figure 1:**
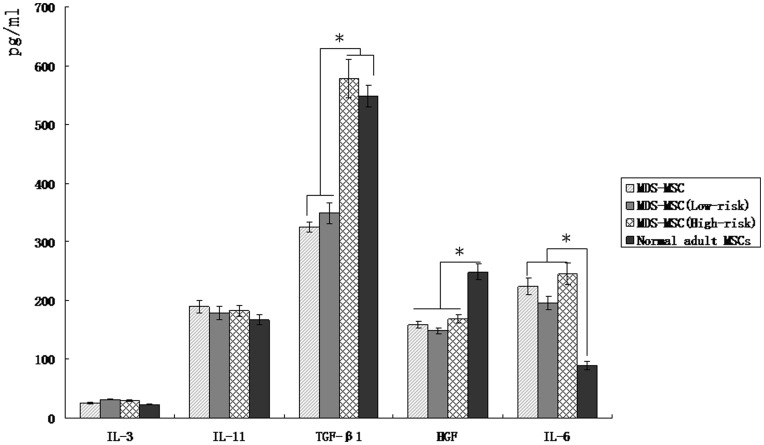
Cytokine secreted by MDS-MSC. The cytokine profiles of normal-MSC, MDS-MSC, high-risk MDS-MSC and low-risk MDS-MSC were assayed by ELISA. Data are expressed as mean±SD of triplicates of 4 separate experiments. *P≤0.05.

Previous studies demonstrated that MSC take part in the regulation of immune by secreting various kinds of cytokines [8]. Using ELISA, our results showed that clonal MDS-MSC secreted more IL-6 but less TGF-β1 and HGF, compared to normal-MSC. The secretion of IL-3 and IL-11 was largely unchanged, as shown in Figure 1. In addition, our results demonstrated that there were great differences between high-risk MDS-MSC and low-risk MDS-MSC in the secretion of cytokines. Compare to high-risk MDS-MSC, low-risk MDS-MSC secreted decreased TGF-β1. The secretion of other cytokines was almost unchanged between high-risk MDS-MSC and low-risk MDS-MSC. Moreover, our data found that the secretion of TGF-β1 was similar between high-risk MDS-MSC and normal-MSC (Figure 1).

### Effect of MSC on T Cell Apoptosis

**Figure 2:**
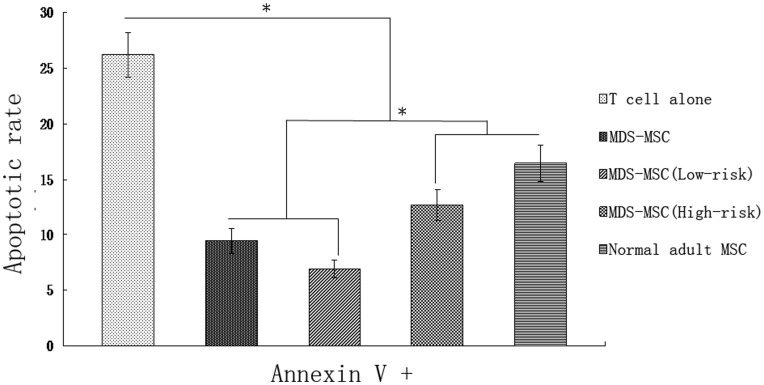
Effect of MDS-MSC on T cell apoptosis. T cells were incubated for 3 days alone or with MDS-MSC or normal-MSC in the presence of the mitogen PHA.The test was conducted by Annexin-V and PI double staining and analyzed by flow cytometry. Data are expressed as mean±SD of triplicates of 5 separate experiments. Annexin V+ means the cells were PI negative and Annexin V positive. *P≤0.05.

To test whether MSC was involved in the regulation of T cells apoptosis, T cells were analyzed for apoptosis after culture alone or coculture with MSC. Our results showed that MSC could significantly decrease the effect of activation-induced apoptosis of T cells. By day 3, the percentage of apoptosis was 26.18±1.98% of T cells in cultures without MSC. However, when cocultured with normal-MSC, the percentage of apoptotic T cells decreased to 16.39±1.65%. In addition, when cocultured with MDS-MSC, the percentage of apoptotic T cells further decreased to 9.48±1.14% (compared with co-culture system of normal-MSC, p<0.05). The next examination was to test whether there were differences in the regulation of T cells apoptosis between high-risk MDS-MSC and low-risk MDS-MSC. The data demonstrated that high-risk MDS-MSC exhibited lower inhibition effect on T cells apoptosis (12.65±1.37%) than low-risk MDS-MSC (6.94±0.82%, p<0.05) (Figure 2). This implies that there are great difference between high-risk MDS-MSC and low-risk MDS-MSC in the inhibited effect on activation-induced T cells apoptosis.

### Inhibitory Effect of MDS-MSC on T Cell Proliferation

**Figure 3:**
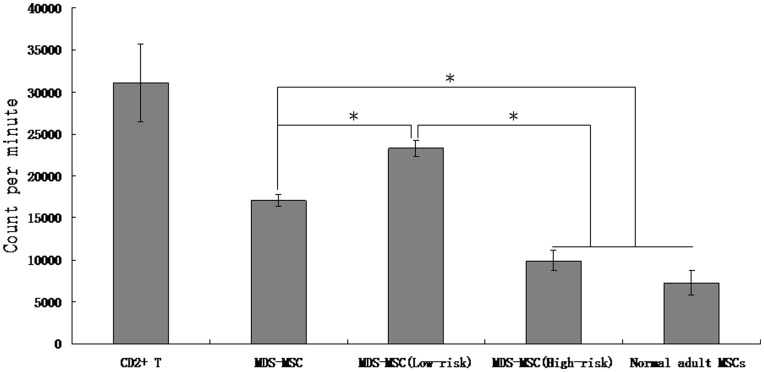
MDS-MSC inhibit T-lymphocyte proliferation. Irradiated (15 Gy) MDS-MSC or normal-MSC were cultured for 5 days with CD2+ T-lymphocyte in the presence of PHA, then assessed by [3H]-thymidine incorporation. Data are expressed as mean±SD of triplicates of 5 separate experiments. *P≤0.05.

In this study, our results demonstrated that MDS-MSC could obviously inhibit the proliferation of T cell stimulated by mitogen. As shown in Figure 3, there was a significant reduction in T cell proliferation when mixed culture of T cell stimulated by PHA was performed in the presence of irradiated MSC (MSC/T-lymphocyte ratio was 1∶10). However, the immunosuppressive rate of MDS-MSC on T cell proliferation was obviously less than that of normal-MSC (45.3±2.3% in MDS-MSC versus 77.5±4.8% in normal derived MSC, p<0.05), suggesting that the immunosuppressive effects of MDS-MSC were impaired.

Given that high-risk MDS-MSC and low-risk MDS-MSC might exist differences in immunoregulatory function, we examined the immunoregulatory function of high-risk MDS-MSC and low-risk MDS-MSC. We found that although both high-risk MDS-MSC and low-risk MDS-MSC could obviously inhibit the proliferation of T cell, the immunosuppressive rate of low-risk MDS-MSC on T cell proliferation was obviously less than that of high-risk MDS-MSC (24.9±1.8% in low-risk MDS-MSC versus 68.4±4.3% in high-risk MDS-MSC, p<0.05). Moreover, the immunosuppressive rate of high-risk MDS-MSC on PHA-induced T cell proliferation was slightly lower than that of normal-MSC (68.4±4.3% in high-risk MDS-MSC versus 77.5±4.8% in normal derived MSC), but not statistically significant (p>0.05) (Figure 3).

### MDS-MSC Induce CD4+CD25+Foxp3+Tregs

**Figure 4:**
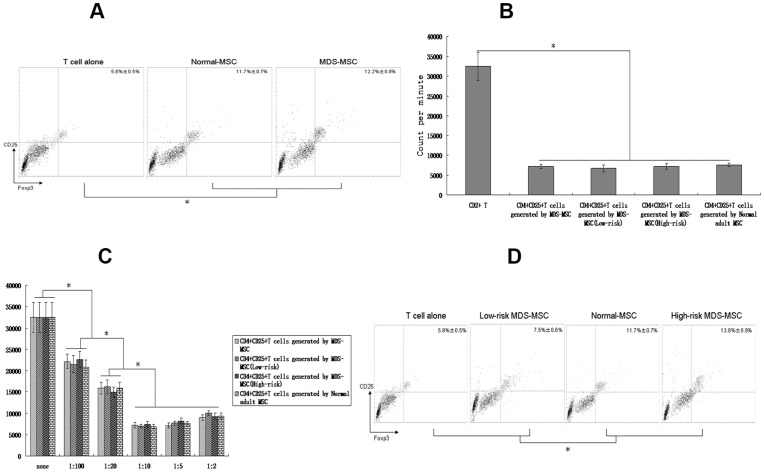
MDS-MSC induce CD4+CD25+Foxp3+Tregs. (A) CD4+CD25-T cells were cultured with MDS-MSC or normal-MSC for 5 days, and CD4+ T cells were collected. The expression of CD25 and Foxp3 on CD4+ T cells was analyzed by FACS. Results are expressed as mean±SD of triplicates of 4 separate experiments. *P≤0.05. (B) CD4+T cells were cocultured with MDS-MSC generated CD4+CD25+Foxp3+Tregs or normal-MSC generated CD4+CD25+Foxp3+Tregs in the presence of PHA, and the T-lymphocyte proliferation was measured on day 5 by [3H]-thymidine incorporation. Results are expressed as mean±SD of triplicates of 4 separate experiments. *P≤0.05. (C) MDS-MSC generated CD4+CD25+Foxp3+Tregs or normal-MSC generated CD4+CD25+Foxp3+Tregs inhibited the response of allogeneic T-lymphocyte in a dose-dependent manner. Responder CD2+ T-lymphocyte were stimulated with PHA for 5 days with or without graded dosed of MDS-MSC generated CD4+CD25+Foxp3+Tregs or normal-MSC generated CD4+CD25+Foxp3+Tregs. Results are expressed as mean±SD of triplicates of 4 separate experiments. *p≤0.05. (D) CD4+CD25-T cells were cultured with high-risk MDS-MSC or low-risk MDS-MSC for 5 days, and CD4+ T cells were collected. The expression of CD25 and Foxp3 on CD4+ T cells was analyzed by FACS. Results are expressed as mean±SD of triplicates of 4 separate experiments. *P≤0.05.

T regulatory cells (Tregs) play an important role in the control of immune reactivity against self-antigens and non-selfantigens [14]. Previous studies had reported that MSC could expand CD4+CD25+Foxp3+Tregs *in vivo* and *in vitro* [15]–[16]. In this study, we cocultured MDS-MSC or normal-MSC with CD4+CD25+T cells, and found that MDS-MSC or normal-MSC could not expand CD4+CD25+T cells *in vitro*. we next wanted to know whether MSC could generate CD4+CD25+Foxp3+Tregs from CD4+CD25-Foxp3-T cells, we cocultured MSC with CD4+CD25-T cells. The results showed that both MDS-MSC and normal-MSC could efficiently generate CD4+CD25+Foxp3+Tregs from CD4+CD25-T cells (5.8%±0.5% for T cell alone, 12.2%±0.8% for MDS-MSC, and 11.7%±0.7% for normal-MSC) (Figure 4A). In addition, the CD4+CD25+Foxp3+Tregs inducible rate of MDS-MSC was slightly higher than that of normal-MSC, but not statistically significant (p>0.05). Our results also demonstrated that both MDS-MSC and normal-MSC induced CD4+CD25+Foxp3+Tregs were able to inhibit T-cell proliferation (Figure 4B). Moreover, we found that the addition of MDS-MSC induced CD4+CD25+Foxp3+Tregs or normal-MSC induced CD4+CD25+Foxp3+Tregs to T-lymphocyte stimulated with PHA suppressed the mitogenic response in a dose-dependent fashion (Figure 4C). At last, our results found that there were great differences in the CD4+CD25+Foxp3+Tregs inducible rate between high-risk MDS-MSC and low-risk MDS-MSC. Compared to high-risk MDS-MSC (13.8%±0.9%), the inducible rate of low-risk MDS-MSC (7.5%±0.6%) was lower (Figure 4D).

### Induction of Tregs by MDS-MSC is Dependent on TGFβ1

**Figure 5:**
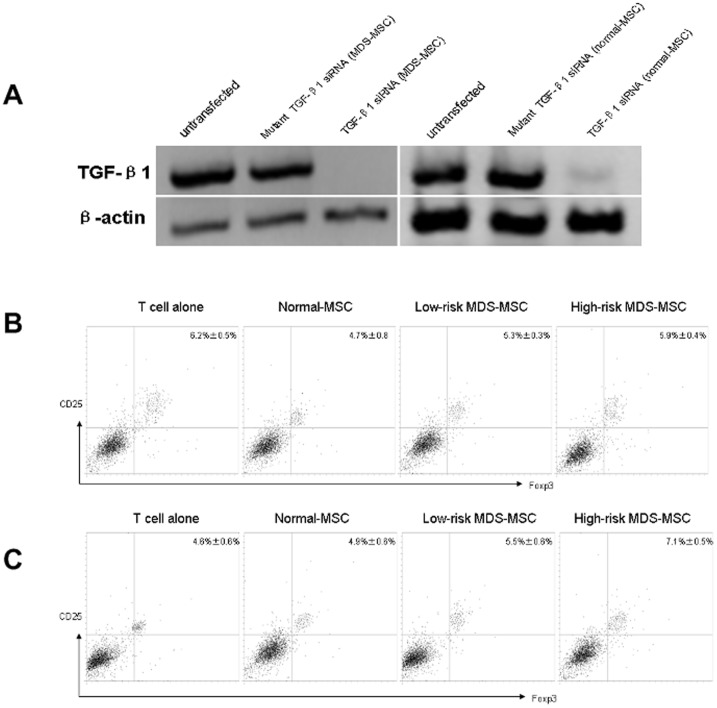
Induction of CD4+CD25+Foxp3+Tregs by MDS-MSC is dependent on TGFβ1. Western blot confirmed efficient knockdown of TGF-β1. (B) CD4+CD25-T cells were cultured with TGF-β1 knockdown MDS-MSC or normal-MSC for 5 days, and CD4+ T cells were collected. The expression of CD25 and Foxp3 on CD4+ T cells was analyzed by FACS. Results are expressed as mean±SD of triplicates of 5 separate experiments. *P≤0.05. (C) CD4+CD25-T cells were cultured with mutant siRNA transfected MDS-MSC or normal-MSC for 5 days, and CD4+ T cells were collected. The expression of CD25 and Foxp3 on CD4+ T cells was analyzed by FACS. Results are expressed as mean±SD of triplicates of 6 separate experiments. *P≤0.05. (D) anti-rhTGF-β1 mAb was added at the beginning of coculture of CD4+CD25-T cells and untransfected MDS-MSC or normal-MSC for 5 days, and CD4+ T cells were collected. The expression of CD25 and Foxp3 on CD4+ T cells was analyzed by FACS. Results are expressed as mean±SD of triplicates of 6 separate experiments. *P≤0.05.

Previous studies showed that TGF-β1 was important cytokine in the induction of tolerance and Tregs [16]–[18]. Based on the data in Figure 2A, we wanted to determine whether MDS-MSC or normal-MSC derived TGF-β1 could induce CD4+CD25+Foxp3+Tregs from CD4+CD25-Foxp3-T cells. In this study, we performed knockdown of TGF-β1 in MDS-MSC or normal-MSC before adding them to cocultures. Knockdown MDS-MSC or normal-MSC were studied for TGF-β1 production. Western blot showed nearly undetectable TGF-β1 in TGF-β1siRNA-transfected MDS-MSC or normal-MSC (Figure 5A). In addition, in the absence of TGF-β1 in MDS-MSC or normal-MSC, there were no significant difference in the generation of CD4+CD25+Foxp3+Tregs (6.2%±0.5% for T cell alone, 5.3%±0.3% for low-risk MDS-MSC, 5.9%±0.4% for high-risk MDS-MSC, and 4.7%±0.8% for normal-MSC) (Figure 5B). These results were the opposite with studies using untransfected MDS-MSC or untransfected normal-MSC (Figure 4A) or mutant siRNA, which showed a significant increase in the generation of CD4+CD25+Foxp3+Tregs. Moreover, anti-rhTGF-β1 was added in cocultures of CD4+CD25-T cells and untransfected MDS-MSC or normal-MSC. We found that there were no significant difference in the generation of CD4+CD25+Foxp3+ Tregs (4.6%±0.6% for T cell alone, 5.5%±0.6% for low-risk MDS-MSC, 7.1%±0.5% for high-risk MDS-MSC, and 4.9%±0.6% for normal-MSC) (Figure 5C). These results indicate a significant role for TGF-β1 secreted by MDS-MSC or normal-MSC in the generation of CD4+CD25+Foxp3+Tregs.

## Discussion

The pathophysiology of MDS involves both intrinsic (cytogenetic, epigenetic) and extrinsic (extracellular, microenvironment) factors. For one thing, the etiology of MDS is presumed that targeted injury or mutation within hematopoietic progenitor cells (intrinsic abnormality) is an early, initiating event in a multistep pathogenesis; For the other, several defects in the bone marrow microenvironment that contribute to the pathogenesis of MDS include the aberrant secretion of angiogenic cytokines by monocyte and stromal cells (or MSC), and the aberrant immunoregulatory functions of MSC.

In addition, current knowledge indicates that aberrant immune responses and T-cell-mediated inhibition of hematopoiesis have been associated with the pathophysiology of MDS. In addition, previous studies have shown that MSC are capable of inhibiting T cells proliferation *in vitro* and mediate a systemic immunosuppressive property *in vivo* [7]–[10]. However, the definitive immunoregulatory function of MDS-MSC and the role of MDS-MSC in the pathogenesis of the disease are extremely limited. For this reason, the investigation of MDS-MSC not only help us to further elucidate etiology and pathology of MDS, but also help us to find new therapeutic strategies for the treatment of MDS.

Previous studies on the immunoregulatory function of MDS-MSC have been controversial [11], [19]–[20]. Klous et al found that MDS patient-derived MSC were capable to sufficiently inhibit T cell proliferation *in vitro* induced by PHA or IL-2 [19]. However, Han et al demonstrated that the immunomodulatory ability of MDS-MSC, compared to MSC from healthy volunteers, was impaired, which might be a cause for an abnormal hematopoietic environment [20]. Consistent with the results of Han’s study, our previous study also found that MDS-MSC showed reduced hematopoiesis support function, as compared to their normal countparts. In addition, the capacity of MDS-MSC to inhibit T lymphocyte activation and proliferation was impaired *in vitro* [11].

Because low-risk MDS and high-risk MDS possess different biological characteristics, including the immunoregulatory functions, we wonder whether there are differences in the immunoregulatory functions between low-risk and high-risk MDS-MSC. In addition, we found that the immunoregulatory functions were investigated in MSC not only derived from low-risk MDS, but also derived from high-risk MDS in Klaous’s study [19]. But, in our and Han’s studies, only low-risk MDS-MSC was investigated for their immunoregulatory functions [11], [20]. So, we hypothesize that the reason of those controversial results is that there are differences in the immunoregulatory functions between low-risk and high-risk MDS-MSC. Moreover, in previous studies, MSC have been analyzed as a complex network of different cell types and molecules, thus it has been difficult to identify and characterize the cell types that are altered in MDS. In order to address these issues, in the present study, we investigate the functional properties of MSC derived from high-risk and low-risk MDS at single cell level.

In this study, MDS-MSC displayed abnormal immunomodulatory function not only in inhibiting activation-induced apoptosis of T cells, but also in inhibiting the proliferation of T cell. In addition, there were several differences between low-risk and high-risk MDS derived MSC. Firstly, our data demonstrated that high-risk MDS-MSC exhibited lower inhibition effect on T cells apoptosis than that of low-risk MDS-MSC; Secondly, we found that the immunosuppressive rate of low-risk MDS-MSC on T-cell proliferation was obviously less than that of high-risk MDS-MSC (24.9±1.8% in low-risk MDS-MSC versus 68.4±4.3% in high-risk MDS-MSC, p<0.05); Thirdly, our results demonstrated that there were great differences between high-risk MDS-MSC and low-risk MDS-MSC in the secretion of cytokines. Analysis of cytokine profiles revealed that high-risk MDS-MSC secreted TGF-β1 at a higher levels than low-risk MDS-MSC; Lastly, the ability of hematopoietic support of high-risk MDS-MSC was significantly less than that of low-risk MDS-MSC (Data not shown). All these results imply that there are great difference between high-risk MDS-MSC and low-risk MDS-MSC in the immunoregulatory functions.

Previous studies demonstrated that the immunomodulatory ability of aplastic anemia (AA) derived MSC, compared to MSC from healthy volunteers, was impaired, which might be a cause for an abnormal hematopoietic environment [21]. Similar to AA, abnormality in the immune system is one characteristic of MDS. Immune mechanisms contribute to the cytopenias of MDS by adversely affecting progenitor survival [22]. In a subset of patients with MDS, laboratory data have shown that myelosuppression is mediated by T lymphocytes [23]–[24]. In this study, although MDS-MSC could significantly decrease the effect of activation-induced apoptosis of T cells, MDS-MSC exhibited lower inhibition effect on T cells apoptosis than that of normal-MSC, implying an overactivated immune reaction in MDS. Moreover, immunomodulatory agents, such as thalidomide, lenalidomide, CSA and ATG, are clinically effective for some MDS cases [3]–[4]. Based on this evidence, normal derived MSC transplantation might be an effective treatment for MDS by utilizing MSC to modulate immune reactions and improve the bone marrow microenvironment.

Tregs maintain immunologic self-tolerance and also suppress immune responses to tumors, transplants, and infectious agents. Previous studies has clearly established that Tregs are increased in human solid tumors as well as hematologic malignancies [25]. In addition, Kordasti et al demonstrate a significant increase in the number of CD4+CD25+Foxp3+Tregs in high-risk MDS [26]. However, little is known about the mechanisms leading to this increase. In this study, our results showed that both MDS-MSC and normal-MSC could efficiently generate CD4+CD25+Foxp3+Tregs from CD4+CD25-T cells. Our finding might be one possible mechanism responsible for expansion of Treg cells in MDS patients. Although Patel et al [27] found that MSC derived from normal adult could efficiently generate CD4+CD25+Foxp3+ Tregs from CD4+CD25-Foxp3-T cells, it was unclear whether this characteristic of MSC were altered in diseased states. In this study, we found that there was a big difference between normal-MSC and MDS-MSC in the role of generation of CD4+CD25+Foxp3+ Tregs. Our result showed that the CD4+CD25+Foxp3+ Tregs inducible rate were significantly higher in high-risk MDS-MSC compared with normal-MSC and in normal-MSC compared with low-risk MDS-MSC and in high-risk MDS-MSC compared with low-risk MDS-MSC. Moreover,CD4+CD25+FOXP3+ Treg cells induced by MDS-MSC were functional and able to suppress T-cell responses. These results suggest that high-risk MDS-MSC may inhibit effective immune responses against the dysplastic clone by inducing CD4+CD25+Foxp3+Tregs, thereby facilitating disease progression. Moreover, low numbers of Tregs induced by low-risk MDS-MSC may permit the emergence of autoreactive T-cell clones and secondary bone marrow hypoplasia.

TGF-β1 is a protein that controls proliferation, cellular differentiation, and other functions in most cells [28]–[29]. TGF-β1 also plays a role in immunity and cancer [30]–[32]. Our previous studies demonstrated that MSC could inhibit T cell proliferation by secreting TGF-β1 [33]. Previous studies also demonstrated that TGF-β1 has been linked to the expansion of Tregs [15]–[16]. In addition, Patel et al demonstrated that MSC derived TGF-β1 could expand CD4+CD25+Foxp3+Tregs [27]. Consistent with this, our results showed that T-lymphocyte proliferation suppressed by MDS-MSC could be restored by high dose of anti-rhTGF-β1 (≥0.5 µg/mL), indicating the inhibitory effects of MDS-MSC were mediated by soluble factor of TGF-β1. Moreover, our results indicate a significant role for TGF-β1 secreted by MDS-MSC or normal-MSC in the generation of CD4+CD25+Foxp3+Tregs based on knockdown studies.

To date, allogeneic hematopoietic stem cells transplantation (allo-HSCT) is still the most effective treatment for MSD. Previous studies demonstrated that MSC in allo-HSCT recipients were damaged and remain of host origin [34]. Therefore, the impaired immunoregulatory and hematopoietic support functions of MDS-MSC may decrease the effectiveness of the therapy of allo-HSCT. Instead, cotransplantation of hematopoietic stem cells and cultured MSC might be a better option to treat MDS patients. Furthermore, our results demonstrated the different immunoregulatory role of MSC in low-risk and high-risk MDS, which may be important for understand the pathogenesis of MDS and the development of novel immunomodulatory strategies for the treatment of MDS. For example, the reduced Tregs frequencies observed in low-risk disease may contribute to the immune-mediated apoptosis. Therefore, cellular therapy based on adoptive transfer of ex vivo expanded Tregs is a potential strategy to treat autoimmune characteristics in low-risk MDS.

Several previous studies suggest a significant deregulation of the immune system in the complex pathogenesis of MDS [1]–[2]. This deregulation may even promote the progression of early MDS to advanced MDS. Previous studies also found that different immune cells, such as DCs, Tregs, NK/T cells, Th17 and cytotoxic CD8+ T cells, were significantly aberrant in patients with MDS [6]. MSC have also been shown to exert a potent suppression on both innate and adaptive immunity by acting on NK and B lymphocytes and DCs and Tregs. In this study, we only demonstrated the different immunoregulatory role of MSC in low-risk and high-risk MDS on T lymphocytes. The next test, focusing on the effect of MDS-MSC on onther immune cells, such as DCs, Tregs, NK/T cells and so on, is necessary.

In conclusion, we investigate for the first time the immunoregulatory functions of MSC in both low- and high-risk MDS at single cell level. Our data demonstrate that there is a big difference in the immune abnormality between low-risk and high-risk MDS-MSC. Compare to low-risk MDS-MSC, high-risk MDS-MSC is associated with the presence of increased Tregs, increased TGF-β1, higher apoptosis, higher immunosuppressive rate and a poor ability of hematopoietic support. In addition, our results demonstrate that MDS-MSC derived TGF-β1 is largely responsible for the increase in CD4+CD25+Foxp3+Tregs based on knockdown studies. Knowing the different immunoregulatory functions between low- and high-risk MDS-MSC may be important for reasonable MSC selection in stem cell based therapy. As a corollary, this would allow an improved exploitation of MSC in other diseases with immune abnormalities.
